# Supplementary figures and images for: Obeticholic acid treatment ameliorates the cardiac dysfunction in NASH mice
Source: PLoS One. 2022 Dec 9;17(12):e0276717. doi: 10.1371/journal.pone.0276717 (PMC9733885; doi:10.1371/journal.pone.0276717)

**Fig. 6A in Figures of manuscript**

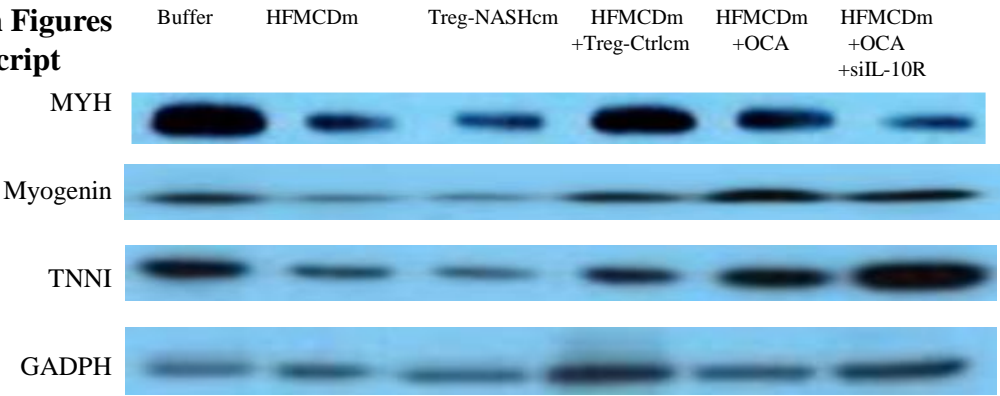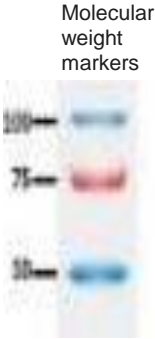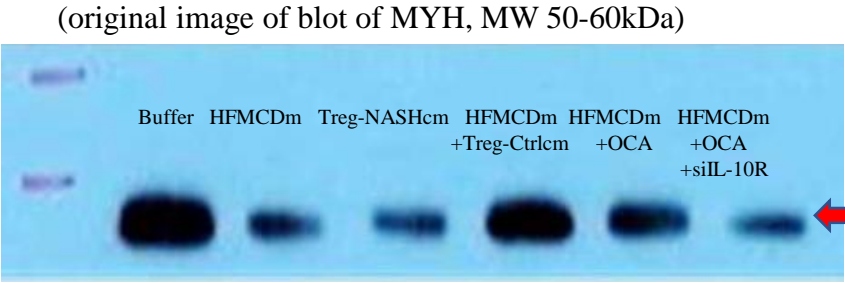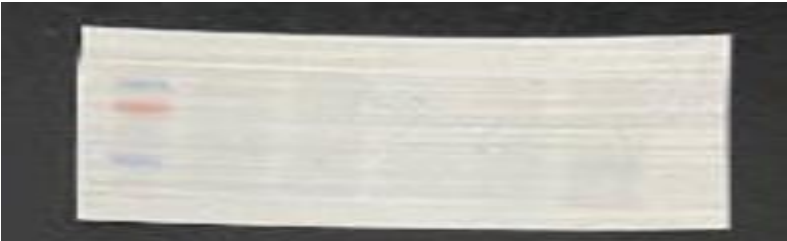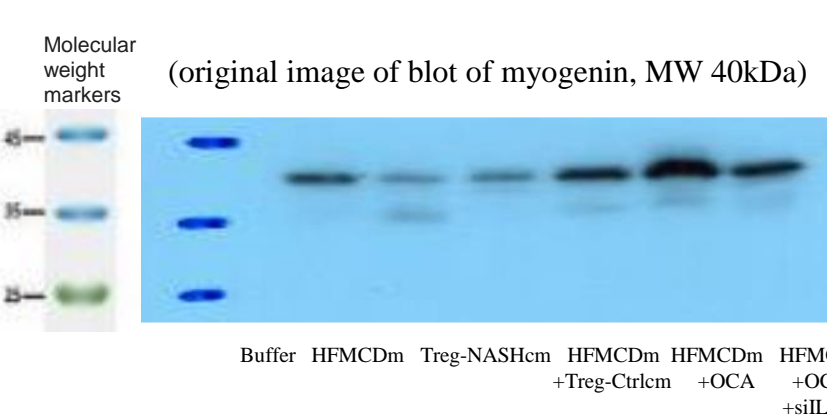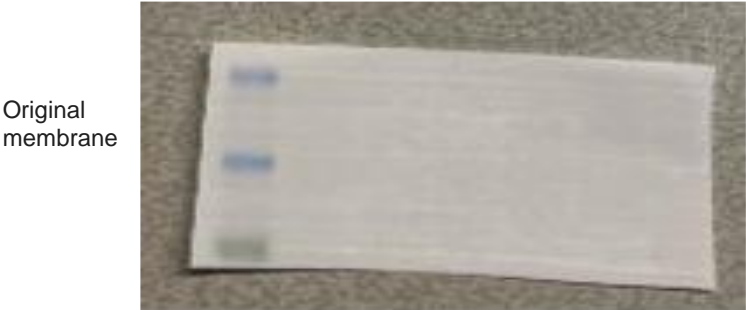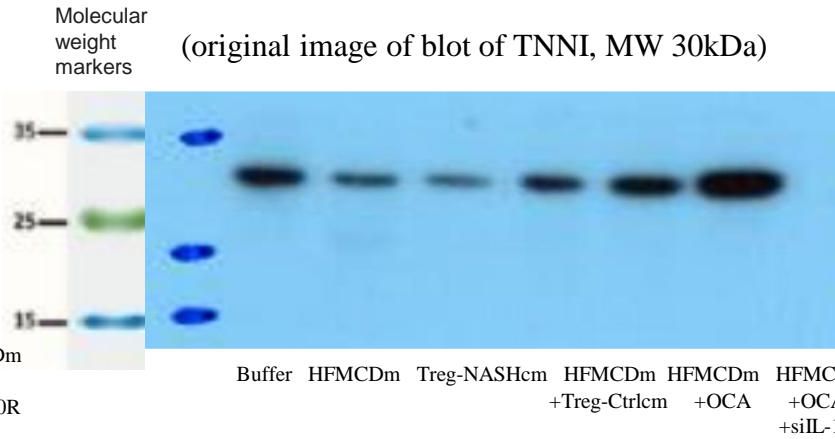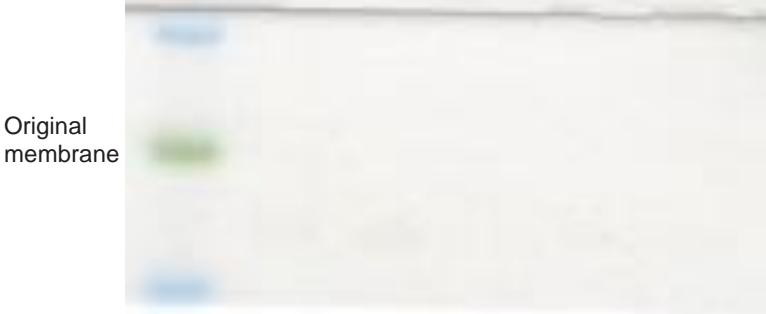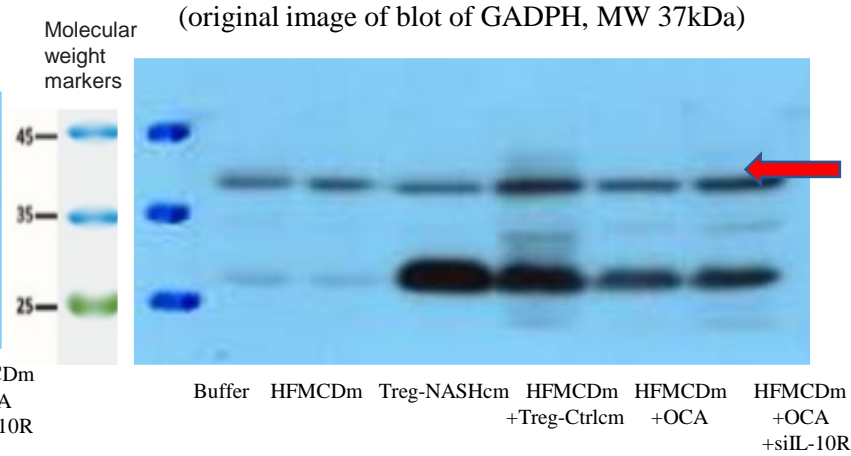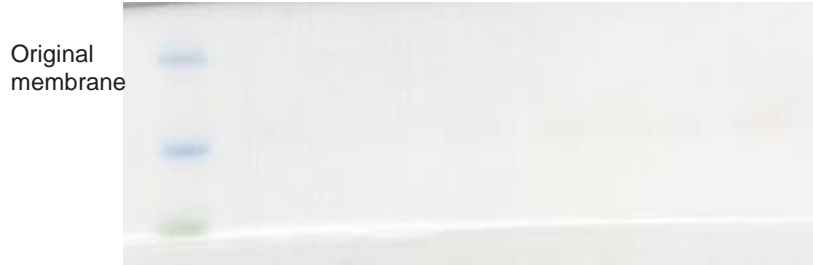

Supplement: S1 Raw images — (PDF) [file pone.0276717.s002.pdf]
