# Supplementary material for: Obeticholic acid treatment ameliorates the cardiac dysfunction in NASH mice
Source: PLoS One. 2022 Dec 9;17(12):e0276717. doi: 10.1371/journal.pone.0276717 (PMC9733885; doi:10.1371/journal.pone.0276717)
Supplement: Attachment — Submitted filename: plos_one_R2_p-t-p-r1006.docx [file pone.0276717.s003.docx]

**PONE-D-22-19785R1**

**Obeticholic acid treatment ameliorates the cardiac dysfunction in NASH mice**

**PLOS ONE
Response to editor office comments:
Thank you for submitting your manuscript to PLOS ONE. After careful consideration, we feel that it has merit but does not fully meet PLOS ONE’s publication criteria as it currently stands. Therefore, we invite you to submit a revised version of the manuscript that addresses the points raised during the review process.**
-**Comment 1**: **Acronyms should be written in extended form the first time they are reported (for example, what "FXR" stands for is not explained in the revised Abstract.**
**Response 1:** Thanks for editor broad team for giving us this opportunity to revise this work. In “revised” abstract and whole manuscript, the full name of acronyms that appearing first time had been included.

Reviewers' comments:

**Comments to the Author**
**Reviewer #1 comments:** The authors have addressed the reviewer's comments and the manuscript has been substantially improved.

**Response to reviewer 1 comment:** Thanks for your very constructive comments to improve our manuscript.

**Reviewer #2 comments:The Authors have revised and improved the manuscript following the Reviewers’ comments.** I have just one further minor suggestion:
**Comment 1**- **Acronyms should be written in extended form the first time they are reported (for example, what "FXR" stands for is not explained in the revised Abstract)**

**Response 1:** Thanks for editor broad team for giving us this opportunity to revise this work. In “revised” abstract and whole manuscript, the full name of acronyms that appearing first time had been included.
